# Susceptibility‐guided sequential strategy versus empirical therapy for *Helicobacter pylori* infection: study protocol for a randomised controlled trial

**DOI:** 10.1186/s13063-023-07457-z

**Published:** 2023-06-19

**Authors:** Kemei Lu, Cuicui Lang, Xuefei Zou, Lina Zang, WeiWei Sang, Qian Feng, Ying Mu, Lifeng Liu, Chunhong Xu, Jingrun Zhao

**Affiliations:** Department of Gastroenterology, Liaocheng People’s Hospital, No. 67 DongchangXi Road, Liaocheng, Shandong Province China

**Keywords:** *Helicobacter pylori*, Randomised controlled trial, Drug resistance, Microbial, Microbial sensitivity tests, Therapeutics

## Abstract

**Background:**

New treatment strategies are required against infections caused by *Helicobacter pylori,* which grows increasingly resistant to antibiotics. Polymerase chain reaction-based methods for antibiotic susceptibility testing are available for detecting *H. pylori*-specific mutations that confer resistance to clarithromycin and levofloxacin. Several meta-analyses have compared eradication rates for susceptibility-guided versus empirical therapy for *H. pylori* treatment; however, all have significant limitations and high heterogeneity, and the results are contradictory. The main objective of this trial is to assess whether a sequential strategy based on molecular susceptibility testing-guided therapy for *H. pylori* has a better eradication rate than empirical therapy.

**Methods:**

This trial is designed as a prospective, randomised, open-label, active-controlled and single-centre study. Men and women who are *H. pylori*-positive, naïve to treatment, and aged 18–65 years will be recruited. A total of 500 participants will be randomised to receive either empirical therapy or a susceptibility-guided sequential strategy. Bismuth quadruple therapy will be the empirical first-line therapy, and in case of failure, high-dose dual (proton-pump inhibitor + amoxicillin) treatment will be the rescue therapy. For the susceptibility-guided sequential strategy, regimen selection will be based on *H. pylori* susceptibility to clarithromycin (first-line) and levofloxacin (rescue). A first-line treatment of clarithromycin triple therapy will be selected for clarithromycin-sensitive strains. For clarithromycin resistance, a high-dose dual therapy will be selected. During the rescue treatment, a levofloxacin quadruple regimen will be selected for levofloxacin-sensitive strains, and a furazolidone quadruple regimen will be selected for others. The primary outcome is the first-line eradication rate in both groups, and the overall (including first and rescue therapies) *H. pylori* eradication rate in both groups is one of the secondary outcomes. The eradication rates of *H. pylori* will be analysed by intention-to-treat analysis, modified intention-to-treat analysis, and per-protocol analysis.

**Discussion:**

This randomised controlled trial will provide objective and valid evidence about the value of polymerase chain reaction-based molecular methods for antibiotic susceptibility testing in guiding *H. pylori* eradication.

**Trial registration:**

Clinicaltrials.gov NCT05549115. Released on 18 September 2022. First posted on 22 September 2022. Enrolment of the first participant on 20 September 2022. The study is retrospectively registered.

**Supplementary Information:**

The online version contains supplementary material available at 10.1186/s13063-023-07457-z.

## Administrative information

Note: The numbers in curly brackets in this protocol refer to the SPIRIT checklist item numbers. The order of the items has been modified to group similar items (see http://www.equator-network.org/reporting-guidelines/spirit-2013-statement-defining-standard-protocol-items-for-clinical-trials/).

**Table:** 

Title {1}	Susceptibility‐guided sequential strategy versus empirical therapy for *Helicobacter pylori* infection: study protocol for a randomised controlled trial
Trial registration {2a and 2b}	Clinicaltrials.gov NCT05549115. Susceptibility‐Guided Sequential Therapy Versus Empirical Therapy for *Helicobacter pylori* Infection: A Randomised Controlled Trial
Protocol version {3}	Protocol version 2.0, 18 October 2022
Funding {4}	Youth scientific research fund of Liaocheng People’s Hospital, No. LYQN202206
Author details {5a}	All authors are affiliated with Liaocheng People’s Hospital.
Name and contact information for the trial sponsor {5b}	Trial sponsor: Liaocheng People’s Hospital Contact name: Mr. Guangyao Li Address: No. 67 Dongchangxi Road, Dongchangfu District, Liaocheng, Shandong Province, China Telephone: + 86–635-827–1235 E-mail: lcrmyy@163.com
Role of sponsor {5c}	The sponsor (i.e., Liaocheng People’s Hospital) will not be involved in any of the study procedures (e.g., study design; collection, management, analysis, and interpretation of data; writing of the report; and the decision to submit the report for publication). The sponsor does not have any authority over any of the above activities.

## Introduction

### Background and rationale {6a}

The pooled *Helicobacter pylori* prevalence is 44.2% (95% confidence interval [CI] [43.0–45.5]) in China, where approximately 589 million individuals are infected with *H. pylori* [1]. *H. pylori* infection is an infectious disease with an indication for antimicrobial therapy [2]. Only a few antibiotics (such as amoxicillin, clarithromycin, metronidazole, tetracycline, levofloxacin, and rifabutin) can be effectively used for the eradication of *H. pylori* in clinical practice. These treatments comprise combination therapies constituting two or three antibiotics, an acid inhibitor, and/or a bismuth component that provides additional antibiotic effects [3]. However, the increasing resistance of *H. pylori* to antibiotics has become a great concern. In a large study conducted in the US and Europe, of 907 participants, 22.2% were resistant to clarithromycin, 1.2% to amoxicillin, and 69.2% to metronidazole [4]. The resistance of *H. pylori* to antibiotics has decreased the eradication rate and caused treatment failure. For example, clarithromycin resistance was found to be significantly associated with the failure of clarithromycin-containing regimens (odds ratio: 6.97; 95% CI [5.23–9.28]; *P* < 0.001) [5]. This situation highlights the need for antibiotic resistance surveillance and the challenge of choosing optimised antibiotics based on antibiotic susceptibility testing (AST).

According to the map of antibiotic resistance of *H. pylori* in China—released by the Institute for Infectious Disease Control and Prevention, China Centre for Disease Control and Prevention (https://map.hla.cn/ )— among the 1,314 reported cases from Shandong Province in 2020, the resistance rates to clarithromycin, metronidazole, and levofloxacin were 65.14%, 94.52%, and 59.89%, respectively. In contrast, resistance to amoxicillin, tetracycline, and furazolidone was 0.30%, 0.00%, and 0.00%, respectively. A meta-analysis of primary antibiotic resistance to *H. pylori* in the Asia–Pacific region reported that the overall mean prevalence of primary *H. pylori* resistance was 17% (95% CI [15-18]) for clarithromycin, 44% (95% CI [39–48]) for metronidazole, 18% (95% CI [15-22]) for levofloxacin, 3% (95% CI [2-5]) for amoxicillin, and 4% (95% CI [2-5]) for tetracycline. The prevalence of resistance to clarithromycin and levofloxacin rose markedly over time during the investigative period, whereas resistance to other antibiotics remained stable [6]. A meta-analysis that reported the resistance patterns of *H. pylori* strains in the United States between 2011 and 2021 found that metronidazole, levofloxacin, and clarithromycin resistance rates each exceeded 30%, whereas resistance to tetracycline, rifabutin, and amoxicillin remained relatively low [7]. The efficacy of clarithromycin- and quinolone-containing regimens is highly affected by their resistance, and repeating these drugs in rescue treatments is discouraged [8, 9]. Therefore, it may be assumed that AST yields useful information regarding only clarithromycin and quinolone in clinical practice.

Choosing an antibiotic regimen with knowledge of the likely pattern of antibiotic resistance is generally preferred. However, a randomised control trial (RCT) recently reported that AST-guided therapy was not superior to personal medication history-guided therapy as a second- or third-line treatment for *H. pylori* infection [10]. Due to its cost-effectiveness, personal medication history-guided therapy is more clinically favourable than AST. A meta-analysis suggested that susceptibility-guided therapy (SGT) may be slightly superior to empirical first-line triple therapy; however, SGT does not appear to be superior to empirical first-line quadruple therapy or empirical rescue therapy [11]. Another meta-analysis revealed that, compared with bismuth-containing quadruple therapy (BQT), SGT showed similar efficacy to the first-line treatment of *H. pylori* infection in areas with high antibiotic resistance [12]. A limitation of RCTs is that many comparative studies evaluating SGT versus empirical treatment included susceptibility testing for only one antibiotic (clarithromycin); metronidazole susceptibility was assessed only in some cases, and quinolone resistance was only exceptionally evaluated [9]. Another limitation is the poor quality of clinical trials using unoptimised regimens and incomparable comparisons related to marked geographic heterogeneity [13]. A meta-analysis reported that the overall eradication rate in patients harbouring susceptible strains was 95.0% (95% CI [94.1–95.9]), but only 63.4% of treatment arms (64/101) achieved good eradication rates (≥ 90%) [14]. Furthermore, almost every RCT study on AST of *H. pylori* only included either first-line or rescue therapies [11, 12, 14–16].

In summary, although SGT is frequently recommended for *H. pylori* infection, the evidence available to date supporting this strategy is limited. Susceptibility testing alone seems insufficient to reliably attain high *H. pylori* eradication rates. The true utility of AST and its timing—before the first treatment or only after eradication failure—are both controversial.

In our study design, AST guides both the first-line and rescue therapies. A sequential strategy should focus not only on the first-line treatment, but also on a consecutive rescue treatment regimen after first-line failure. At the same time, the choice of treatment regimens should be based on local susceptibility, updated guidelines, and recent RCT results [17–20]. Clarithromycin resistance is the main factor to consider when deciding which regimen to use for *H. pylori* infections; thus, for the first-line treatment, we chose therapy regimens based on clarithromycin susceptibility. After first-line treatment failure, fluoroquinolone-containing quadruple (or triple) therapy may be recommended, as per the Maastricht VI/Florence consensus report [17]. Therefore, for the rescue treatment, we chose therapy regimens based on levofloxacin susceptibility. We hypothesise that, compared with empirical therapy, the susceptibility-guided sequential strategy will demonstrate a higher eradication rate for *H. pylori* infection.

## Objectives {7}

The main objective of this trial is to assess whether a sequential strategy based on molecular AST for *H. pylori* infection will have a better eradication rate than an empirical therapy (ET).

## Trial design {8}

**Fig. 1:**
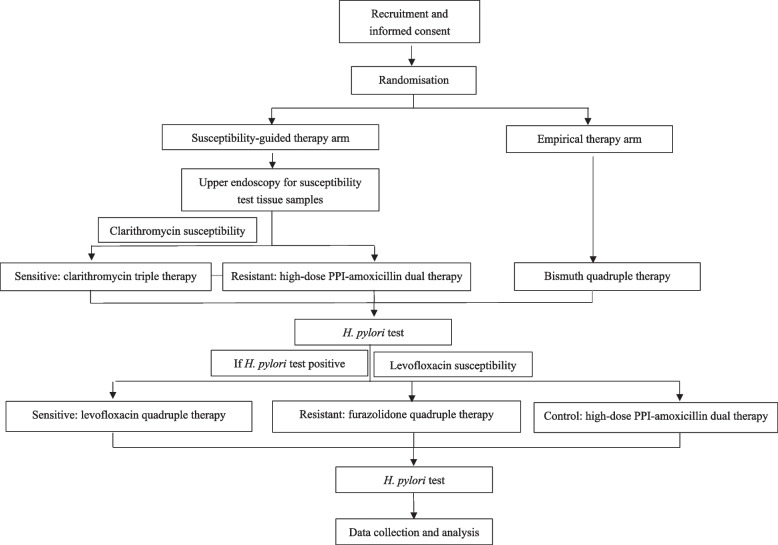
Flowchart of the study. PPI: Proton-pump inhibitor

This trial is designed as a prospective, single-centre, randomised, open-label, active-controlled equivalence study with a 1:1 allocation ratio. The study procedure is detailed in Fig. 1.

## Methods: participants, interventions, and outcomes

### Study setting {9}

The study will be conducted primarily in the outpatient clinic and endoscopic centre of Liaocheng People’s Hospital, which is a Class A comprehensive tertiary hospital in China.

### Eligibility criteria {10}

#### Inclusion criteria

1. 18–65 years of age (including men and women).
2. *H. pylori*-positive, which will be determined using at least one of the following tests: 13C/14C-urea breath test (UBT), stool *H. pylori* antigen test, rapid urease test, and histological analysis within 4 weeks.
3. Naïve to *H. pylori* treatment.

#### Exclusion criteria

1. Allergic to any drug administered.
2. Pregnant or lactating.
3. Has major systemic diseases, such as severe cardiopulmonary or liver dysfunction.
4. Diagnosed with active peptic ulcer disease within the past 8 weeks, history of gastric cancer, or prior gastrectomy.

### Who will take informed consent? {26a}

Participants must provide written informed consent before any study procedures occur. The investigators will explain and discuss the trial with the potential participants, confirm that they understand the research, and ensure that their agreement to participate is voluntary. The investigators will obtain written informed consent from the participants.

### Additional consent provisions for collection and use of participant data and biological specimens {26b}

Gastric mucosal samples will be stored for use in future studies. The original informed consent form includes information on data collection and request. Consent will be obtained from participants to use their data and specimens in future research unrelated to the clinical condition under study.

## Interventions

### Explanation for the choice of comparators {6b}

The ET group will receive BQT as a first-line treatment because the combination of amoxicillin and clarithromycin (74.8%) is the most commonly prescribed treatment in China [21] and has a high eradication rate for naïve patients. Moreover, antimicrobial resistance does not markedly affect its therapeutic efficacy [22]. The BQT regime comprises esomeprazole (20 mg, twice daily), colloidal bismuth pectin (150 mg, four times daily), clarithromycin (500 mg, twice daily), and amoxicillin (1.0 g, twice daily) taken for 14 days. If BQT fails, high-dose dual therapy (HDDT)—esomeprazole (20 mg, four times daily) and amoxicillin (750 mg, four times daily)—will be used as a rescue treatment for another 14 days. This regime was recommended by the Maastricht VI/Florence consensus [17]. Additionally, meta-analyses have reported that the HDDT regimen has better efficacy and safety in *H. pylori* eradication than current guideline-recommended regimens, particularly in Asia [20, 23]. The HDDT regimen does not rely on a susceptibility test, as the resistance rate to amoxicillin is extremely rare (< 5%), even after the failure of treatment, allowing it to be empirically prescribed.

### Intervention description {11a}

The SGT group’s regimens will be selected according to *H. pylori* susceptibility to clarithromycin and levofloxacin. Participants will undergo an upper endoscopy to provide gastric mucosal samples for molecular testing. The test is based on polymerase chain reaction (PCR), which detects *H. pylori* mutations associated with clarithromycin and levofloxacin resistance. The susceptibility testing PCR protocol is outlined in Additional File 1. Regimens for the first-line treatments are based on clarithromycin susceptibility: If the *H. pylori* strain is sensitive to clarithromycin, we will administer clarithromycin triple therapy—esomeprazole (20 mg, twice daily), clarithromycin (500 mg, twice daily), and amoxicillin (1.0 g, twice daily)—for 14 days, as per the recommended guideline [17]. If the strain is clarithromycin resistant, we will administer the HDDT regimen because a network meta-analysis revealed that this treatment is the most optimal first-line therapy for *H. pylori* among the Asian population [24]. If the first-line treatment fails, rescue therapy will be selected according to levofloxacin susceptibility. If the *H. pylori* strain is levofloxacin sensitive, we will administer the levofloxacin quadruple regimen—esomeprazole (20 mg, twice daily), levofloxacin (500 mg, once daily), amoxicillin (1.0 g, twice daily), and colloidal bismuth pectin (150 mg, four times daily)—for 14 days, as per the recommended guideline [17]. If *H. pylori* is levofloxacin-resistant, we will administer the furazolidone quadruple regimen—esomeprazole (20 mg, twice daily), furazolidone (100 mg, twice daily), amoxicillin (1.0 g, twice daily), and colloidal bismuth pectin (150 mg, four times daily)—for 14 days. This furazolidone regimen can provide a satisfactory eradication rate in patients with multiple treatment failures and does not increase the incidence of adverse events [25, 26].

### Criteria for discontinuing or modifying allocated interventions {11b}

The safety of *H. pylori* eradication treatments is well defined, where taste disturbance, diarrhoea, nausea, and abdominal pain are the most frequent adverse events (AEs). The majority of AEs are mild and temporary. It is estimated that only 1.3% of patients discontinue treatment due to AEs [27]. In our study, when participants report AEs, they will be evaluated by the investigators. The study drugs may be discontinued for any of the following reasons: participants experience severe AEs, are declared unsuitable for continued drug use, or cannot comply with the study procedures. Participants may withdraw from the study for any reason at any time. The reasons for withdrawal will be recorded if disclosed by the participants.

### Strategies to improve adherence to interventions {11c}

Face-to-face adherence reminder sessions will take place during the initial drug dispensing. Sessions will cover the importance of participants completing treatment regimens and calling investigators if experiencing problems that may be related to study drugs. Moreover, participants will receive instructions on taking study pills (dose, timing, storage) and what to do in the event of a missed dose. Furthermore, participants will receive a compliance reminder phone call 1 week after the start of treatment, and a 13C-UBT test reminder call 4 weeks after the end of treatment.

### Relevant concomitant care permitted or prohibited during the trial {11d}

During the study procedure, the use of all other antibiotics, proton-pump inhibitors (PPIs), H2 receptor antagonists, nonsteroidal anti-inflammatory drugs, Chinese traditional herbs, and probiotics will be prohibited.

### Provisions for post-trial care {30}

No specific post-trial care is planned, as the study is a low-risk intervention. The study site has insurance to cover for harms associated with the trial. This includes cover for additional health care, compensation, or damages.

### Outcomes {12}

The primary outcome of the study is the proportion of participants with successful *H. pylori* eradication. This will be determined according to the results of 13C-UBTs performed at least 4 weeks after the end of the first-line treatment period between the SGT and ET groups.

The secondary outcomes:

1. The rescue treatment and the overall eradication rates in the SGT and ET groups.
2. The eradication rate of HDDT for the clarithromycin resistance group and the empirical BQT failure group.
3. Frequency of AEs and the rate of compliance between the SGT and ET groups.

### Participant timeline {13}

**Fig. 2:**
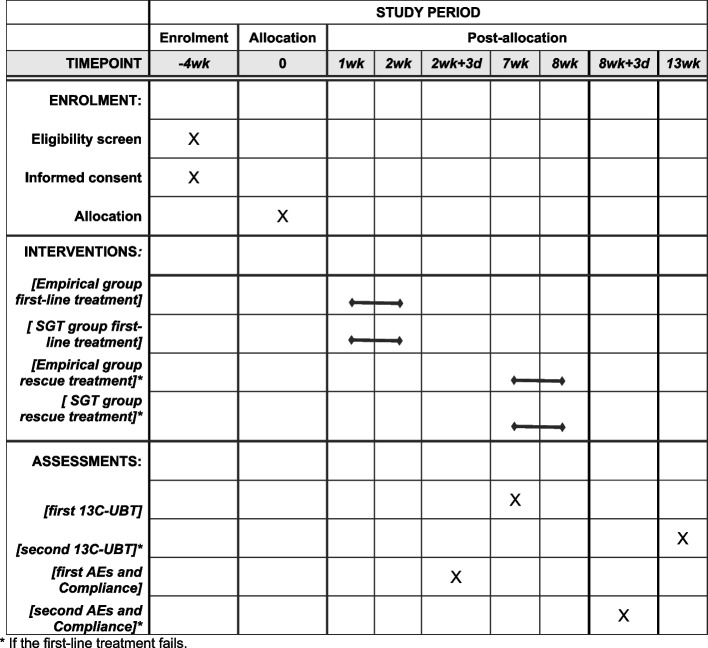
Study schedule of enrolment, intervention, and assessments. AEs: Adverse events; SGT: Susceptibility-guided therapy; UBT: Urea breath test

The schedule of enrolment, interventions, and assessments are summarised in Fig. 2.

### Sample size {14}

We will conduct this as an equivalence study, and the calculation of the sample size is based on the primary outcome, which is the first-line eradication rate of *H. pylori*. Using intention-to-treat (ITT) analysis, a meta-analysis comparing SGT vs. BQT as the first-line treatment for *H. pylori* infection found that the pooled eradication rates of SGT and BQT were 86% vs. 78% [12]. A recent meta-analysis reported that the overall eradication rate in patients harbouring susceptible strains was 95.0% (95% CI [94.1–95.9]), but only 63.4% of treatment arms (64/101) achieved good eradication rates (≥ 90%) [14]. Based on these data, our assumption is a 90% eradication rate of susceptibility‐guided sequential strategy, 80% eradication rate of empiric therapy, power of 80%, alpha of 0.05 (two-sided), and allocation ratio of 1:1. The calculated sample size is 197 participants per arm. Considering a dropout rate of 20%, we have to recruit at least 494 patients (247 participants per arm) for the study. Therefore, we aim to recruit 500 participants for this study.

### Recruitment {15}

Participants will be recruited primarily in the outpatient clinic as well as the endoscopic centre of Liaocheng People’s Hospital by the investigators. In addition, we have a dedicated phone number to answer any inquiries about participation in the study. It is estimated that there are 100–150 recently diagnosed *H. pylori* infection patients visiting our outpatient clinic each month. The investigators will inform eligible patients about the study and ensure that they understand the implications of participating in it. Patients will be asked to give consent to participate in the study by signing an informed consent form.

## Assignment of interventions: allocation

### Sequence generation {16a}

For random allocation, an independent research assistant will generate a randomised number table using IBM SPSS Statistics for Windows software version 22.0 (IBM Corp., Armonk, NY, USA) ahead of the study.

### Concealment mechanism {16b}

The randomised number table will be sealed in an opaque envelope and retained by the independent research assistant.

### Implementation {16c}

Once the investigators obtain informed consent from a participant, they will call the independent research assistant to obtain the allocated regimen for that participant.

## Assignment of interventions: blinding

### Who will be blinded {17a}

As this is an open-label study, it is impossible to blind the participants or investigators. However, the data collectors and data analysts will remain blinded to treatment allocation throughout the study. For AEs and compliance, a data collection form (Additional File 2) concealing the allocation information will be used, and data collectors are not allowed to ask participants about their treatment regimen. The allocation information will also be concealed from the dataset that will be distributed to the data analysts.

### Procedure for unblinding if needed {17b}

Not applicable because it is not possible to blind the participants or investigators.

## Data collection and management

### Plans for assessment and collection of outcomes {18a}

Sociodemographic and baseline information (including name, sex, age, contact information, and past medical history) will be collected during screening. The success of *H. pylori* eradication will be assessed using 13C-UBT 4 weeks after therapy completion. Before getting the 13C-UBT done, participants will not be allowed to use any PPIs or antibiotics for 2 weeks and 30 days, respectively. Considering the impact of the COVID-19 pandemic, we require the 13C-UBT to be done at most 8 weeks after the completion of therapy. The information on AEs and compliance throughout the study will be collected via telephone and recorded using the data collection form (Additional file 2) 3 days after the completion of therapy. Study drug compliance will be based on the number of days of drug administration. Because 10-day therapy regimens can achieve good eradication rates [28, 29], 10 or more days will be considered good compliance and less than 10 days bad compliance. Data will be collected by two dedicated data collectors who will be trained in collecting the information in a uniform, reproducible manner, and the quality of the data will be supervised by the principal investigator.

### *P*lans to promote participant retention and complete follow-up {18b}

Investigators will provide periodic communications with participants via telephone and WeChat. They will use methods to improve participant retention, such as reminders for taking the study drug, and scheduling appointments for the 13C-UBT.

### Data management {19}

Two dedicated investigators will be responsible for data entry and data accuracy by double-checking the data. All data will be entered electronically. The original study forms will provide the option to choose a value from a list of valid codes and a description of what each code means. Checks will be applied at the time of data entry to ensure that the data go into appropriate fields. Participant files are to be stored in numerical order in a secure place. The files will be maintained in storage for a period of 5 years after the completion of the study. Access to the study data will be restricted using a password system.

### Confidentiality {27}

All collected data from this study will be coded using unique patient identifiers so that no individual subjects can be identified. Patient records will be accessed only as per the regulations of the Ethics Committee of the Liaocheng People’s Hospital, and only when necessary. To the extent permitted by applicable laws and regulations, any records relating to participant identification are confidential and will not be made public.

### Plans for collection, laboratory evaluation and storage of biological specimens for genetic or molecular analysis in this trial/future use {33}

The investigators will obtain gastric mucosal biopsy specimens for antimicrobial susceptibility testing. Two biopsy specimens will be obtained from each patient: one from the greater curve of the antrum and one from the lesser curve of the gastric body. The specimens, which are marked with unique patient identifiers, will be kept in only one refrigerator at -28 °C, and maintained by a specimen administrator who will keep the refrigerator key. ShunFeng EXPRESS will transfer the specimens to Zhiyuan Medical Inspection Institute Co., Ltd. (Hangzhou, China) every 3 days using a drikold delivery box. A standard protocol will be used for the biopsy, storage, and transfer processes. The *H. pylori* and genotypic resistance statuses of each patient will be revealed by PCR testing, which will help detect the presence of mutations conferring resistance to clarithromycin and levofloxacin.

## Statistical methods

### Statistical methods for primary and secondary outcomes {20a}

The eradication rate of *H. pylori* will be evaluated using ITT, mITT (including all participants who received at least one dose of the study drug), and per-protocol (PP) analyses (including participants who had good compliance and were re-examined by 13C-UBT). A point estimate and two-sided 95% CI of the difference in eradication rates between the SGT and ET groups will be calculated via the Miettinen & Nurminen method for the primary and secondary outcomes. Participants who have not undergone a re-examined 13C-UBT will be considered as treatment failures, i.e., ‘not eradicated,’ in the statistical analysis. Propensity score analysis will be used to adjust for differences in treatment regimens and potential confounding factors. Compliance and sensitivity analyses will be conducted to compare the treatment responses between compliant and non-compliant participants and determine if there is a significant association between compliance and treatment outcomes. Statistical significance will be set at *P* < 0.05.

### Interim analyses {21b}

Not applicable; no interim analysis is planned.

### Methods for additional analyses (e.g., subgroup analyses) {20b}

We plan to conduct a subgroup analysis that compares the *H. pylori* eradication rate between the clarithromycin resistance group and the empirical BQT failure group. Both groups are HDDT treatment regimens; the former is first-line treatment, and the latter is the rescue treatment. We anticipate that HDDT will be equally effective in both groups. A point estimate and a two-sided 95% CI of the difference in eradication rates between the two groups will be calculated via the Miettinen & Nurminen method. Statistical significance will be set at *P* < 0.05.

### Methods in analysis to handle protocol non-adherence and any statistical methods to handle missing data {20c}

The primary and secondary outcomes will be evaluated using ITT, mITT, and PP analyses. Participants who have not undergone a re-examined 13C-UBT will be considered as treatment failures, i.e., ‘not eradicated,’ in the statistical analysis. Strategies will be implemented to maximise follow-up, improve adherence, and prevent missing data. We will report the reasons for non-adherence for each randomisation group and compare the reasons qualitatively.

### Plans to give access to the full protocol, participant-level data, and statistical code {31c}

We will deliver a completely deidentified data set to an appropriate data archive for sharing purposes within 1 year after the study completion.

## Oversight and monitoring

### Composition of the coordinating centre and trial steering committee {5d}

The principal investigator is the designer of the trial and is responsible for conducting the study. The principal investigator and research physicians are responsible for the recruitment, treatment, follow-up of study participants, severe AEs, and serious unexpected suspected adverse events (SUSAR) reports. The Data Manager will be responsible for data collection, data entry, and data verification. A steering committee will be set up to supervise the entire study process. The study team will meet every two weeks to monitor the study’s conduct. Frequent contact via WeChat and telephone will ensure that the study runs smoothly.

### Composition of the data monitoring committee, its role and reporting structure {21a}

No data monitoring committee will be established because the trial has a short duration, and the treatment regimens are associated with known, minimal risks.

### Adverse event reporting and harms {22}

In this study, we will monitor for treatment-emergent AEs, which are defined as any events that occur after the administration of the first dose of a study drug, or any events at baseline that worsen in either intensity or frequency after the first dose of the study drug. An adverse event that meets the criteria for a serious adverse event will be reported to the Ethics Committee of Liaocheng People’s Hospital.

### Frequency and plans for auditing trial conduct {23}

An auditor will review the study processes related to participant enrolment, consent, eligibility, and allocation to study groups; adherence to trial interventions and policies to protect participants, including reporting of harms; and completeness, accuracy, and timeliness of data collection. The auditing will conduct at least one onsite monitoring visit per 3 months over the course of the study. The process of auditing will be independent of investigators and the sponsor.

### Plans for communicating important protocol amendments to relevant parties (e.g., trial participants and ethical committees) {25}

Any modifications to the protocol that may have an impact on the conduct of the study, such as anything to affect the potential benefits and risks, will be approved by the Ethics Committee of Liaocheng People’s Hospital prior to implementation and communicated to the health authorities in accordance with local regulations.

### Dissemination plans {31a}

The study results will be released to the participating physicians, patients, and the general medical community. The results will be disseminated regardless of the magnitude or direction of the treatment’s effect. The findings will be published in peer-reviewed journals and presented at national and international conferences.

## Discussion

Antimicrobial susceptibility tests have been explored to overcome the eradication failure of *H. pylori,* as it gains more antimicrobial resistance to clarithromycin, levofloxacin, and metronidazole. However, RCTs and meta-analyses have revealed contradictory results [11, 12, 14, 16, 30, 31]. Possible factors that may impact eradication rates are the following: *H. pylori* susceptibility, the use of multiple lines of treatment, and combining different treatment regimens. A meta-analysis indicated that the pooled eradication rate of *H. pylori* was 86% when SGT was used for first-line treatments [12]; however, another study reported an eradication rate of 72% when SGT was used in third-line treatments [32]. This shows that an SGT strategy might provide a better eradication rate than empirical methods in first-line treatments but not in second- or third-line treatments [30, 33, 34]. In this study, we applied a sequential strategy based on *H. pylori* susceptibility to clarithromycin and levofloxacin. This promising strategy comprises first-line and rescue treatments, which are based on the most current guidelines and the latest published RCTs on *H. pylori* [17–20].

The use of cultures from gastric biopsies to detect *H. pylori* antibiotic resistance is the gold standard in clinical practice. PCR-based molecular methods for susceptibility testing are suitable for detecting clarithromycin and quinolone susceptibility profiles in *H. pylori*. Moreover, PCR methods are more reproducible, rapid, and cost-efficient than culture-based methods, and are perhaps more suitable for clinical practice [35]. The COVID-19 pandemic resulted in PCR testing being almost universally available at hospitals, which means readily available PCR kits can be repurposed to detect the antibiotic resistance of *H. pylori* rapidly and inexpensively [36].

In general, medication compliance is defined as adherence to taking more than 80% and less than 120% of the treatment drugs [12, 37]. In this study, compliance is defined as patients taking drugs for 10–14 days. This is because RCTs and an international, multicentre, prospective, non-interventional study reported that 10 days of treatment was enough to reach a satisfactory eradication rate [28, 29, 38, 39].

The limitation of our study is that to obtain gastric biopsies for susceptibility testing, we require endoscopies, which are expensive, uncomfortable, and have a low acceptance rate by patients. Further studies into the development of more easily accessible methods of resistance testing, such as stool testing for the six commonly used antibiotics without the need for endoscopy, are required.

In summary, this randomised controlled trial will provide objective and valid evidence about the value of PCR-based molecular methods used for AST in guiding *H. pylori* eradication.

## Trial status

The protocol version is V2.0, 18 October 2022. The first participant was enrolled on 20 September 2022, recruitment is expected to be completed by the end of September 2023, and the trial is estimated to end in December 2023.

## Supplementary Information

**Additional file 1.** Polymerase chain reaction detection of H. pylori infection and mutations conferring resistance to clarithromycin and levofloxacin.

**Additional file 2.** Data collection form.

## Abbreviations

AE: Adverse event
AST: Antibiotic susceptibility testing
BQT: Bismuth-containing quadruple therapy
CI: Confidence interval
ET: Empirical therapy
HDDT: High-dose dual therapy
ITT: Intention-to-treat
PCR: Polymerase chain reaction
PP: Per-protocol
PPIs: Proton-pump inhibitors
RCT: Randomised control trial
SGT: Susceptibility-guided therapy
UBT: Urea breath test
