# Supplementary material for: Susceptibility‐guided sequential strategy versus empirical therapy for Helicobacter pylori infection: study protocol for a randomised controlled trial
Source: Trials. 2023 Jun 19;24:413. doi: 10.1186/s13063-023-07457-z (PMC10278287; doi:10.1186/s13063-023-07457-z)
Supplement: Supplementary file 2 — Additional file 2. Data collection form. [file 13063_2023_7457_MOESM2_ESM.docx]

Data collection form

| Participant No. | Sex: male/female | Date of birth: | |  |
| --- | --- | --- | --- | --- |
|  | Question | Answer | | Remarks |
| Question 1 | Have you completed the therapy period? | Yes | No | If the answer is no, go to question 1a and 1b |
| Question 1a | How many days did you take the medication? |  | |  |
| Question 1b | Why did you not complete the therapy period? |  | |  |
| Question 2 | Did you have any adverse events? |  | |  |
| Sign | Date |  | |  |
